# Embracing the Diversity of Halogen Bonding Motifs in Fragment-Based Drug Discovery—Construction of a Diversity-Optimized Halogen-Enriched Fragment Library

**DOI:** 10.3389/fchem.2019.00009

**Published:** 2019-02-18

**Authors:** Johannes Heidrich, Laura E. Sperl, Frank M. Boeckler

**Affiliations:** ^1^Lab for Molecular Design & Pharmaceutical Biophysics, Department of Pharmacy and Biochemistry, Institute of Pharmaceutical Sciences, Eberhard Karls Universität Tübingen, Tübingen, Germany; ^2^Center for Bioinformatics Tübingen (ZBIT), Eberhard Karls Universität Tübingen, Tübingen, Germany

**Keywords:** fragment, library, HEFLib, design, diversity, V_max_

## Abstract

Halogen bonds have recently gained attention in life sciences and drug discovery. However, it can be difficult to harness their full potential, when newly introducing them into an established hit or lead structure by molecular design. A possible solution to overcome this problem is the use of halogen-enriched fragment libraries (HEFLibs), which consist of chemical probes that provide the opportunity to identify halogen bonds as one of the main features of the binding mode. Initially, we have suggested the HEFLibs concept when constructing a focused library for finding p53 mutant stabilizers. Herein, we broaden and extent this concept aiming for a general HEFLib comprising a huge diversity of binding motifs and, thus, increasing the applicability to various targets. Using the construction principle of feature trees, we represent each halogenated fragment by treating all simple to complex substituents as modifiers of the central (hetero)arylhalide. This approach allows us to focus on the proximal binding interface around the halogen bond and, thus, its integration into a network of interactions based on the fragment's binding motif. As a first illustrative example, we generated a library of 198 fragments that unifies a two-fold strategy: Besides achieving a diversity-optimized basis of the library, we have extended this “core” by structurally similar “satellite compounds” that exhibit quite different halogen bonding interfaces. Tuning effects, i.e., increasing the magnitude of the σ-hole, can have an essential influence on the strength of the halogen bond. We were able to implement this key feature into the diversity selection, based on the rapid and efficient prediction of the highest positive electrostatic potential on the electron isodensity surface, representing the σ-hole, by V_max_Pred.

## Introduction

**Figure 1:**
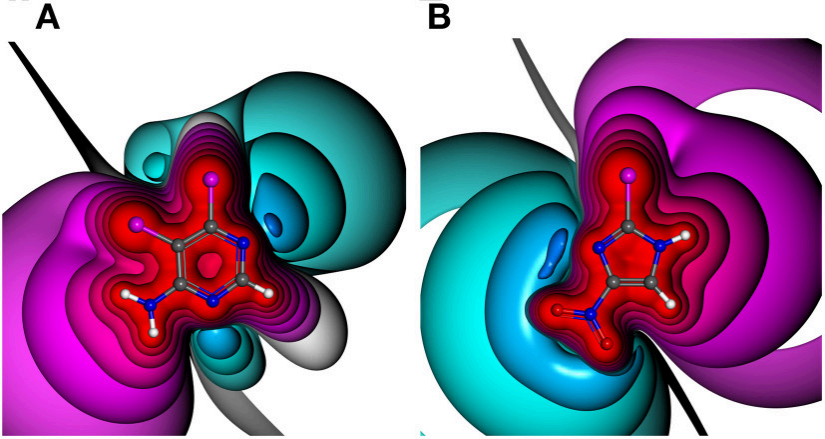
Electrostatic potential (ESP) plots of different halogen-bearing aromatic ring systems with net charge of null. ESP from −0.34 au (dark blue) via zero (white) to +0.34 au (red). Example molecules out of generated HEFLib: **(A)** 5,6-dichloro-4-pyrimidinamine and **(B)** 2-bromo-4-nitro-1H-imidazole.

The manifold and constantly increasing application of halogen bonding (XB) in different areas of life sciences, e.g., biomolecular engineering (Carlsson et al., 2018) and drug discovery (Scholfield et al., 2013; Sirimulla et al., 2013; Zimmermann et al., 2014), emphasizes the demand for a profound understanding of the requirements and versatility of this highly directed molecular interaction. XB as a non-bonded interaction is established by the attraction of a partially positive region at the halogen in extension of the R-X axis, where X is in most cases a chlorine, bromine or iodine atom and R is an electron withdrawing group, with an electron donor moiety, i.e., a π- or n-electrons (Clark et al., 2007; Politzer et al., 2010). Note that also fluorine can undergo halogen bonding in rather rare situations which are unlikely to be observable in drug discovery projects (Metrangolo et al., 2011; Eskandari and Lesani, 2015). This region of positive electrostatic potential is called the σ-hole and can be explained by the electron configuration of the heavier halides $s^{2}p_{x}^{2}p_{y}^{2}p_{z}^{1}$, where the *p*_*z*_ orbital is oriented along the R-X axis. In most cases of simple and symmetric molecules, this positive region is surrounded by a negative belt (Clark et al., 2007). The binding energy of halogen bonding varies between very weak and strong ionically assisted interactions (180 kJ/mol of ionic complexes), depending on the interaction partners (Metrangolo et al., 2005; Domagała et al., 2018). While certainly dependent on the type of the halogen atom and the Lewis base (LB), interaction hotspots can typically be expected at distances *d*_*X*⋯*LB*_ of ~2.75–3.5 Å and σ-hole angles α_*C*−*X*⋯*LB*_ between 155 and 180° (Wilcken et al., 2013). These short optimal distances can be explained by the non-spherically symmetric electron densities of heavy halides, which is described by the term “polar flattening” (Sedlak et al., 2015). The σ-hole can be characterized by its magnitude, size, linearity, and range (Kolár and Hobza, 2016). The magnitude is commonly expressed as the maximum electrostatic potential (ESP) value (called V_S, max_ or V_max_) located on the halogen surface of electron density of 0.001 or 0.002 au (Murray and Politzer, 1991; Riley et al., 2011). At this electron density, 96–97% of the molecular charge is included (Bader et al., 1987; Politzer et al., 2010; Kolár and Hobza, 2016). Different (hetero)aromatic ring systems as well as their substitution patterns have a significant influence on the shape and the magnitude of the σ-hole (see Figure 1) (Lange et al., 2019). This effect is also known as “tuning” (Riley et al., 2011, 2013).

The rather narrow geometric demands for good to optimal halogen bonds can be challenging barriers in the process of molecular design, especially when trying to introduce them into an already established and more complex framework of interactions between a protein and a ligand. To overcome these difficulties and to identify halogen bonding “hot spots” of a protein, while including the advantages of fragment-based drug discovery (FBDD), halogen-enriched fragment libraries (HEFLibs) can be used as a suitable toolkit. This concept was already successfully applied in order to identify p53 mutant stabilizers (Wilcken et al., 2012). The unconventional binding motifs of these fragments resulted in a series of ligands with remarkable affinities. FBDD is driven by the idea to identify molecular hits for a drug target by using less and significantly smaller compounds than these of conventional lead-like or drug-like libraries (Ray et al., 2017). In comparison to different estimates of drug discovery-relevant molecules in chemical space, from 16.6·10^10^ for up to 17 non-hydrogen atoms to 10^60^ for up to 30 non-hydrogen atoms, the size of the chemical space from which typical fragment libraries are selected is much smaller (Bohacek et al., 1996; Ruddigkeit et al., 2012). By applying simple combinatorics it is obvious that the higher the atom count of a molecular graph, the higher the number of molecules that can be generated. In the reverse case, the smaller the number of atoms, the lower the number of possible enumerations. Thus, medium-sized fragment libraries are considered better representatives of their respective chemical space, than large-scale HTS libraries. The binding of a small molecule to a drug target is induced by a number of directed and non-directed interactions. In case of fragments, the number of possible interactions per molecule is smaller than those of drug-like molecules. In line with the concept of “molecular complexity and obesity” (Hann et al., 2001; Hann, 2011), small parts of a binding site can accommodate a fragment with higher ligand efficiency (Keserü and Makara, 2009; Schultes et al., 2010) at the cost of lower absolute affinities. As a consequence, more targets can be addressed resulting in a higher hit rate (Chessari and Woodhead, 2009) of weak binders. To compensate for these low affinities, screening experiments are performed at relatively high concentrations, requiring a high solubility of the fragments (Boyd et al., 2012). By growing or linking small-sized hits to lead structures, new drug candidates with higher affinities can be created (Erlanson, 2011; Trapero et al., 2018). Whereas, drug-like libraries are commonly built with respect to Lipinski's rule of five (Lipinski et al., 2001), for fragment libraries the rule of three (Congreve et al., 2003) is applied, which requires an octanol-water partition coefficient logP ≤ 3, MW ≤ 300 Da, not more than 3 hydrogen bond donors or acceptors and not more than 3 rotatable bonds. Such restriction enforce a small number of interactions per hit (Joseph-McCarthy et al., 2014). Strategic enrichment of a desired interaction type can significantly increase the probability of observing this particular interaction as a key binding motif. Fragment libraries can either be unfocused or they can be designed for addressing serine, cysteine (Backus et al., 2016; Craven et al., 2018) and lysine residues by establishing covalent bonds (Kathman and Statsyuk, 2016) with distinct functional groups like boronic acids, epoxides, acrylamides, or *N*-succinimidyl ester.

In addition, they can be designed to address difficultly shaped binding pockets by a considerable fraction of sp^3^-hybridized moieties. Another possible strategy is to design a fragment library focused on a specific target by including a knowledge-based set of suitable interaction motif into each fragment, e.g., well-known kinase hinge binder (Xing et al., 2015).

Herein, we present a generalized design strategy for halogen-enriched fragment libraries (HEFLibs) as chemical probes for identifying halogen bonds as key binding elements. Tuning effects of halogen atoms connected to (hetero)aromatic ring systems are included as a selection criterion into the feature tree-like (Rarey and Dixon, 1998) diversity assessment aiming for a maximal interface diversity proximal to the halogen bond. By treating each aromatic ring system with a connected heavier halogen, i.e., chlorine, bromine, or iodine, as the central element for molecular similarity assessment and all of its substituents as modifiers, we created a similarity measure that is tailor-made for emphasizing the influence of the local environment of the respective halogen on the possible interaction pattern of this “binding motif.” At the same time, the influence of more halogen-distant molecular features is decreased. This construction principle of a rooted tree, based on the abstraction of the molecular graph, was successfully implemented to predict V_max_ (Heidrich et al., 2018) and is herein utilized for the diversity assessment of halogenated fragments. As a first showcase application, we compiled a library of 198 fragments that consists of a diversity-optimized “core” which is extended by structurally similar, but with regard to their halogen bonding interface quite different, “satellite compounds.” When applying this diversity-optimized HEFLib to a variety of different target proteins, we propose that the experimental results can inspire a better understanding of geometric requirements and tuning effects of halogen bonds. Thus, it can be a useful tool for studying halogen bonding with biochemical, biophysical and structural methods. In addition, the use of our diversity-optimized HEFLib can yield hits with unprecedented binding modes and unique features for patenting.

## Methods

### Diversity Assessment

In contrast to the original implementation of feature trees by Rarey and Dixon (1998) our approach uses a rooted tree, where the aromatic ring with a halogen defines the key property for diversity assessment.

**Figure 2:**
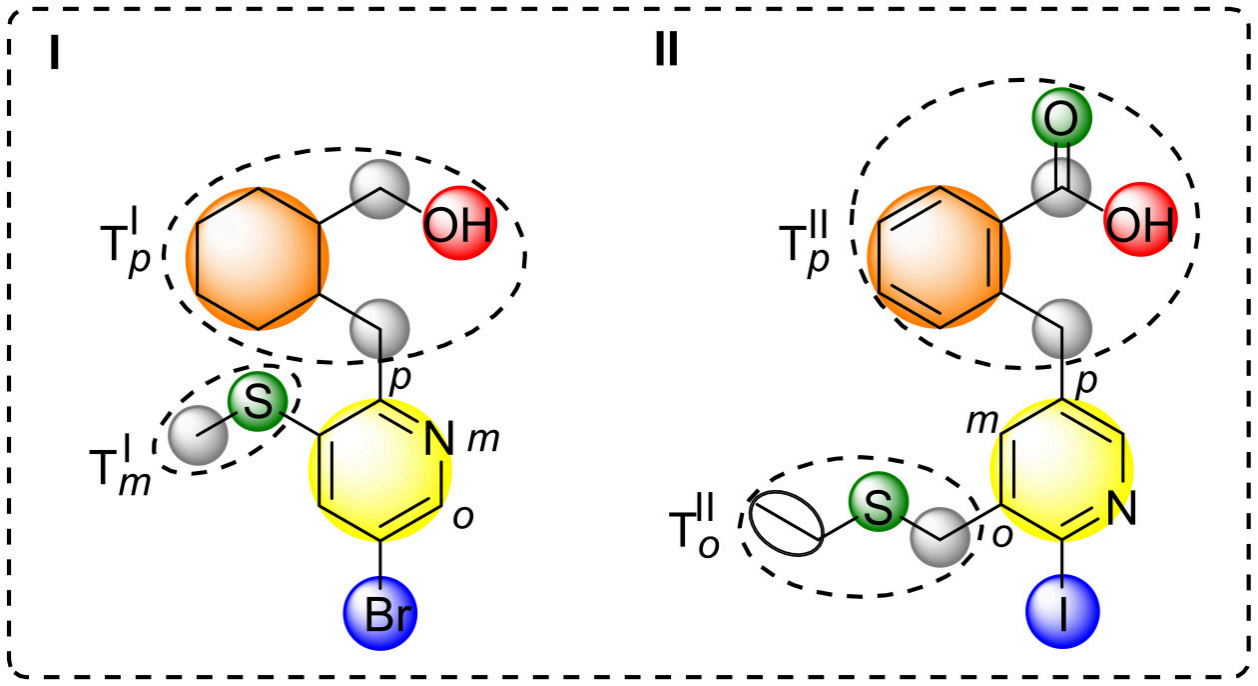
Schematic depiction of feature tree-like comparison of two molecules **(I**,**II)**. Comparison node-based on aromatic ring (yellow) that carries halogen (blue) and defines the central molecular property, hydrogen bond acceptors and donors (green and red), ring structures (orange), and linkers (gray). Rooted comparison of *ortho, meta*, and *para* sub tree of molecule **I** and **II** ($T_{o,m,p}^{I,II}$).

All simple and complex substituents (e.g., single hydrogen atoms or annulated ring systems) connected to this central aromatic ring are treated as residues resulting in a halogen interface-focused diversity measure (see Figure 2). Similarity assessment of two halogenated fragments is done by the pair-wise comparison of their aromatic moieties and their connected residues with regard to the topological constitution. By taking topologically shifted and unmatched residues into consideration, an optimal matching is determined. Each halogen attached to an aromatic ring is considered as a single “configuration” that is pair-wise compared to another molecule's configuration by preserving the nodal matching between the two halogen atoms (for an example see Figure 2). Besides the basic nodal properties of the central aromatic moiety and its attached halogen (see yellow and blue circle in Figure 2), information about charges, i.e., positively and negatively charged atoms, hydrogen bond donors/acceptors, rings, and linkers are included. Thereby defined nodes contain property values of atom count (Todeschini and Consonni, 2009), lone-pair electrostatic interaction (Cheng and Yuan, 2006; Todeschini and Consonni, 2009), information index on proton-neutron composition (Bonchev et al., 1976; Todeschini and Consonni, 2009), kappa shape index-related flexibility index (Kier, 1989; Hall and Kier, 2007; Todeschini and Consonni, 2009), edge connectivity index (Cash, 1995; Estrada, 1995; Todeschini and Consonni, 2009), and group electronegativity (Zhou et al., 2007; Todeschini and Consonni, 2009). Moreover, each node that encodes an aromatic halogen atom contains the information of the predicted V_max_ value at the electron isodensity level of 0.020 au (Heidrich et al., 2018), which was derived by a machine-learned SVM model. Furthermore, atom types and topologies of the central aromatic ring are taken into consideration. Different weighting parameters for feature tree-like comparison were empirically determined by generating small test cases and comparing the output to the desired relative similarity.

### Solubility Prediction

Fragment solubility is one of the key properties for a successful *in vitro* screening, since fragments are known to bind only with a millimolar to high micromolar affinity, screening experiments are conducted under relatively high ligand concentrations. We applied a consensus prediction scheme using six known solubility predictors [AlogPS 2.1 (Tetko et al., 2001; Tetko and Tanchuk, 2002), WSKOWIN 1.24 (U.S. Environmental Protection Agency, 2017), ESOL (Delaney, 2004), MOE (Chemical Computing Group ULC, 2018), QPlogS and CIQPlogS of Schrödinger Suite] of which three need to predict a logS better than −2 in case of a heavy atom count (HAC) small than 16, or better than −1 for fragments with up to 22 heavy atoms. For predicted solubility values see Figure S1.

### Experimental Solubility Assessment

Turbidimetric solubility assessment of fragments available in larger quantities was done in 50 mM HEPES pH 7.0 and 100 mM NaCl at room temperature. Ninety-six-well plate containing ligand stocks in DMSO of 100 mM was diluted by factor 5/6 in DMSO. Diluted compounds were added to buffer resulting in 5% DMSO in 200 μL using VIAFLO 96 automated multi-channel pipette (INTEGRA Biosciences Deutschland GmbH). Measurement at 600–800 nm was done in five kinetic cycles of each 115 s with double orbital shaking at 300 rpm for 60 s before readout using CLARIOstar (BMG Labtech) resulting in a total observation time of approximately 15 min. Since compounds showed no significant absorption at higher dilution levels in this wavelength spectrum, any increase in absorption is assumed to be based on insoluble particles/precipitation of the ligand.

### Filtering

Pricing information was gathered from Aldrich Market Select website and used as hard limit ($5/mg). Compounds without aromatic halogen and larger than 20 heavy atoms were removed from vendor library. Additionally, unwanted and reactive substructures were removed by applying SMARTS filter. All structure filtering steps were done using KNIME (Berthold et al., 2007) and the RDKit (Landrum, 2006).

### Fragment Selection

A set of 150 diverse “core” compounds was selected applying a MaxMin (Ashton et al., 2002) picking scheme with different initially selected compounds from the pair-wise calculated distance matrix of 2,685 compounds of Aldrich Market Select that fulfill the minimum requirements defined above. Additionally, the five most similar “satellite” compounds were denoted and a sub selection was done with expert opinion, based on affordability and predicted solubility, resulting in 200 compounds. By including affordable and highly soluble “satellite” compounds into our library, we aimed to increase the probability of reaching high ligand concentrations in *in vitro* experiments for each of the selected halogen bonding interfaces of the “core” compounds. Due to delivery problems, 2 compounds could not be ordered resulting in the final HEFLib with 198 fragments. The library provided in SMILES codes and respective properties of each fragment can be downloaded as Supplementary Material (Data Sheet 1).

## Results

### The Necessity for an Advanced XB Interface Description

**Figure 3:**
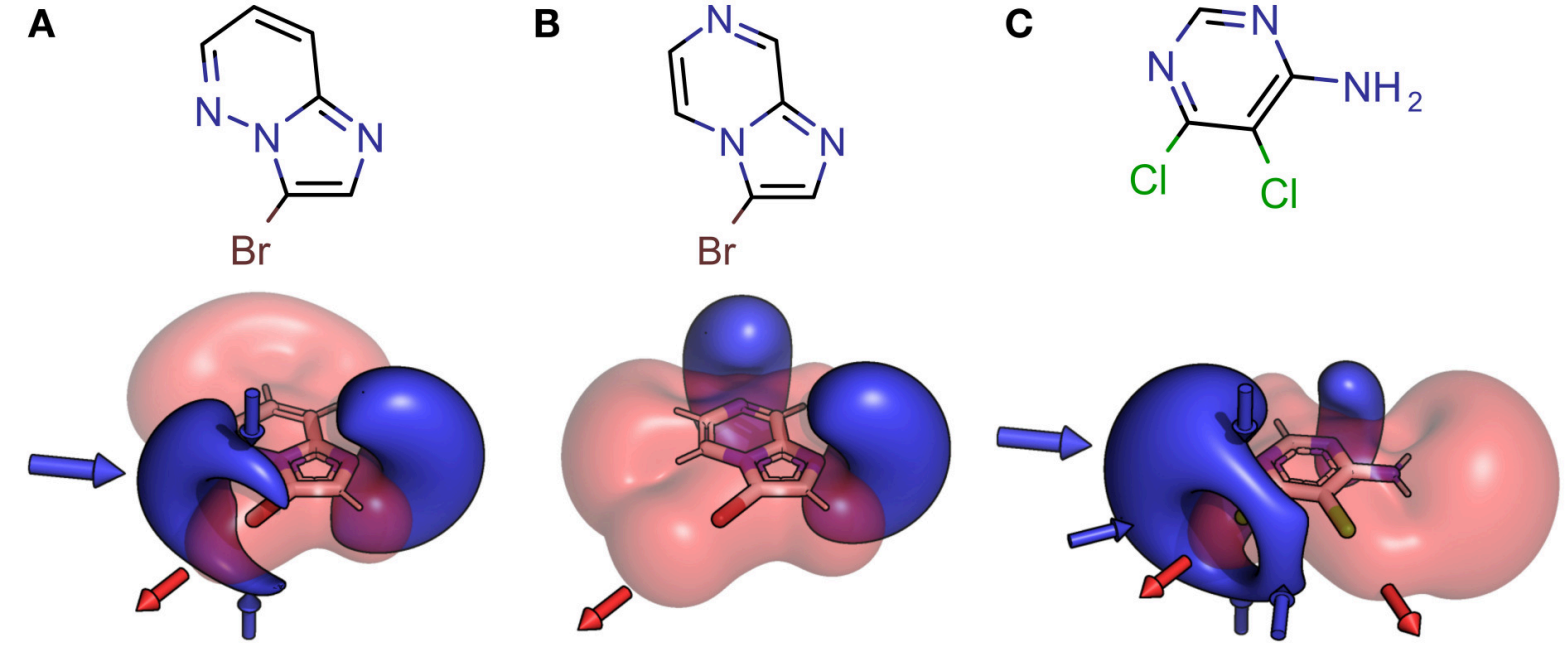
Structural formula and 3D depictions of electrostatic potentials, illustrating similarities and diversities with respect to chemotype and XB interface (binding motif). Three examples from the herein presented showcase HEFLib are shown. Pharmacophoric arrows indicate typical vectors of electrophilic attack toward the ligand (blue) or toward the target by σ-hole interactions (red). Upon shifting the pyridine-type nitrogen atom from position 5 in 3-bromoimidazo[1,2-*b*]pyridazine **(A)** to position 7 in 3-bromoimidazo[1,2-*a*]pyrazine **(B)**, electron density is withdrawn from the negative belt of the halogen toward the opposite direction of the σ-hole. Consequently, a much larger positive electrostatic potential representing a significantly tuned σ-hole with reduced directionality is characteristic for the halogen bonding interface of **(B)**. In case of 5,6-dichloropyrimidin-4-amine **(C)** the same molecule offers one classical XB interface with addressable electron density around the halogen atom and one significantly tuned halogen bonding interface. Despite significant differences in the chemotype, the XB interface of **(A)** and the classical XB interface of **(C)** share some obvious similarities.

Halogen bonding is typically rationalized by considering the electrostatic features of the ligand and target. Still, it should not be forgotten that to some extend induction, dispersion (Politzer et al., 2010; Riley et al., 2013) and charge transfer (Rezáč and de la Lande, 2016) will also play a role in halogen bonding. The electrostatic potential (ESP) (Murry and Politzer, 2011), as a physical observable, was used to define and visualize the XB interface of the fragments in our library. The high directionality of halogen bonding can be explained by the electron anisotropy of heavy halides (chlorine, bromine, and iodine) and their belt of negative electrostatic potential surrounding the σ-hole in case of simple aryl halides. Nevertheless, this symmetric shape can be significantly altered by neighboring groups and heteroaromatic scaffolds, having a strong impact on optimal interaction angles and distances. Our initial, unfocused library design aims at highly diverse XB interfaces, where the halogen bond is embedded in a multitude of chemical environments, leading to variations of the electrostatic features and resulting pharmacophoric interactions with a putative binding site (see Figure 3).

### Characterization of the Herein Selected Showcase HEFLib

**Figure 4:**
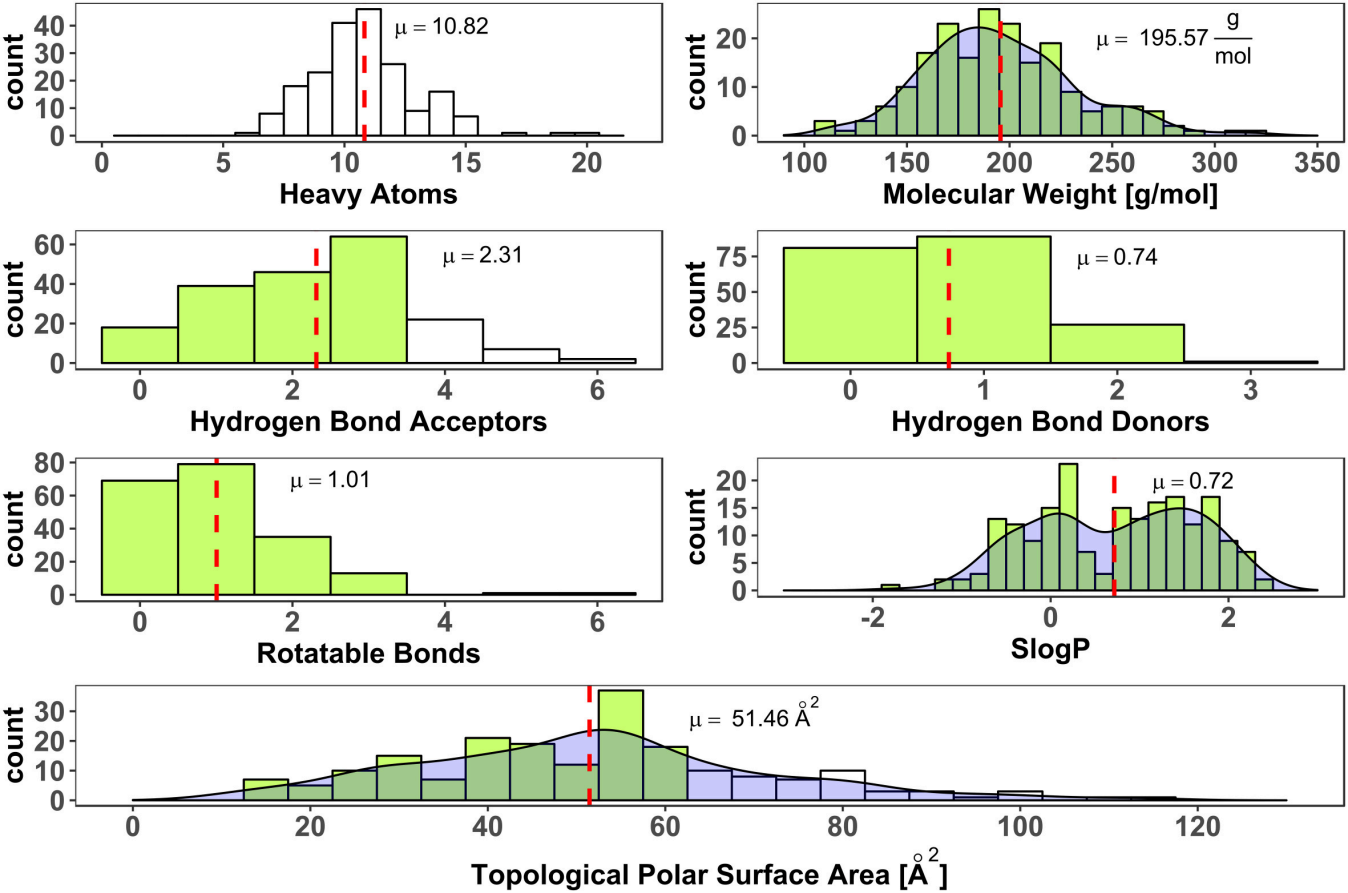
Distribution of number of heavy atoms (RDKit), molecular weight (Canvas Molecular Descriptors, Schrödinger), number of hydrogen bond acceptors (CDK), number of hydrogen bond donors (CDK), number of rotatable bonds (CDK, non-terminal), SlogP as cLogP (RDKit) and Topological Polar Surface Area (Ertl et al., 2000) (RDKit). Dashed red lines indicate mean values (μ). Green bars indicate bins that fulfill rule of three (Congreve et al., 2003). Density shown for continuous data.

To avoid a selection bias due to molecular weight restrictions, no hard limit of molecular weight was included in the process of fragment selection from vendor libraries. Weight and size deviate strongly for the heavy halogens, bromine (equivalent weight to 6.7 carbon atoms), and iodine (equivalent weight to 10.6 carbon atoms). Thus, halogen-enriched fragments are expected to have a larger molecular weight than standard fragments. Nevertheless, only two fragments violated the *rule of three* in terms of molecular weight. Thirty one fragments contain more than three hydrogen bond acceptors. Two compounds have more than three rotatable bonds and 56 have a larger polar surface area (PSA) than 60 Å^2^ (see Figure 4). Both parameters, rotatable bonds and polar surface area, can give a hint for oral availability. High values of these parameters might go hand in hand with high solubility, which we aimed for. Interestingly, linear models were found that show a negative correlation of solubility with topological polar surface area, i.e., with decreasing TPSA aqueous solubility increases (Ali et al., 2012). Iteratively refined values for optimal polar surface area of fragments are described by Ray et al. (2017). Applying their limitation of up to 90 Å^2^ for the PSA, only 9 fragments of the generated HEFLib violate this guideline. Furthermore, they increased the number of favorable hydrogen bond acceptors to 6, which includes all fragments of the generated HEFLib.

### Turbidimetric Solubility Assessment

Depending on the criterion of absorption/extinction threshold for the detection of insoluble compounds, six to nine compounds out of 96 tested fragments showed turbidity in the assay at the highest concentration of 5 mM (Figure S2). Nevertheless, all tested compounds were soluble at the lowest tested concentration of 1.67 mM, except for one compound that was not completely soluble at 100 mM in DMSO. In this case the actual concentration of the dilution series in DMSO remains unclear. It needs to be mentioned, that our assay format is much more sensitive to intrinsic absorption of the compounds than the classical nephelometric turbidity assay. For all measured compounds an absorption spectrum was recorded at a concentration of 1.25 mM. At this concentration, all compounds were soluble. Within the range of 600–800 nm, no significant absorption maximum was observed.

### Three-Dimensionality

**Figure 5:**
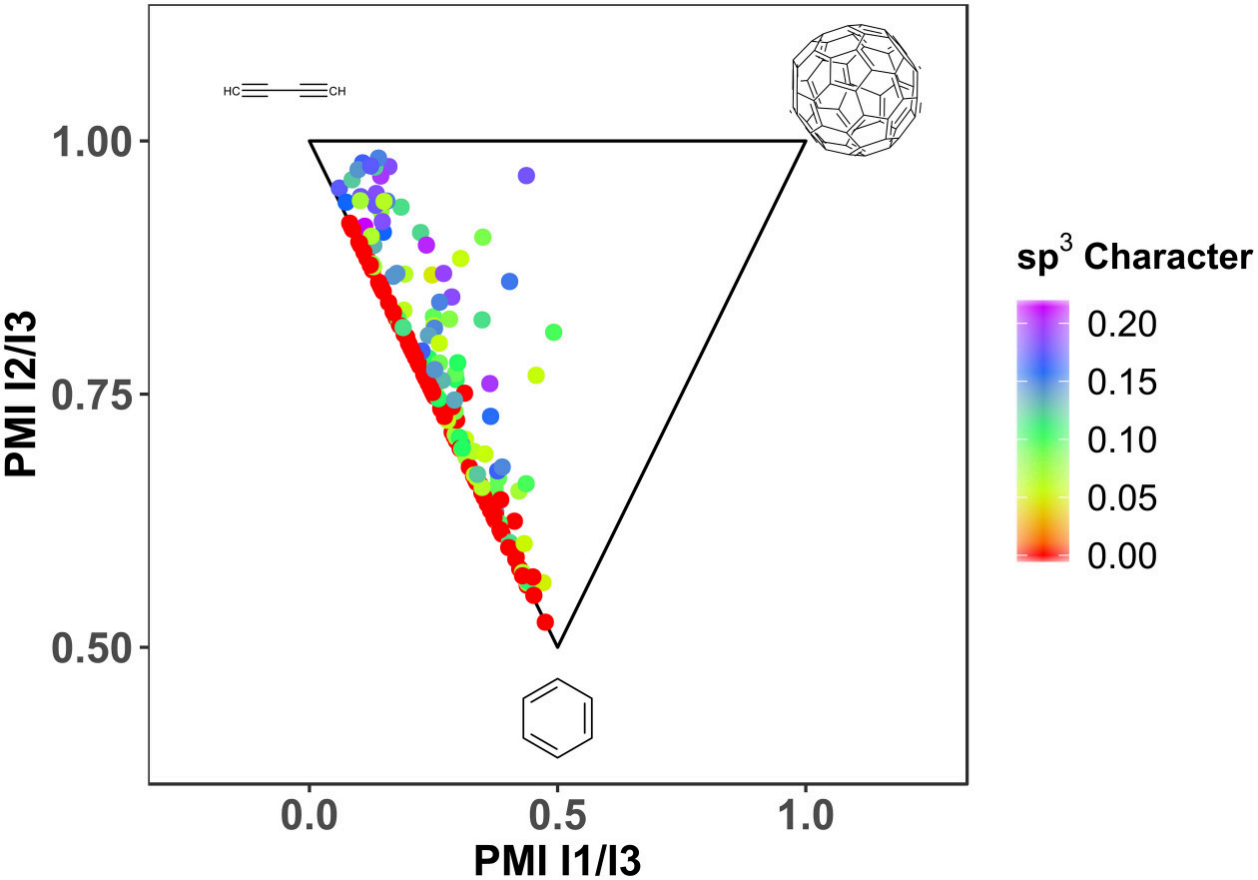
Shape distribution of HEFLib with *n* = 198. Geometry optimization by LigPrep (Schrödinger Release., 2018) (Schrödinger) and OPLS2005. sp3 character calculated with CDK (Steinbeck et al., 2003, 2006; Willighagen et al., 2017). PMI (Sauer and Schwarz, 2003) calculation with Vernalis Nodes for KNIME.

Another important factor for the fragment library design is defined by three-dimensionality (Hung et al., 2011; Bower et al., 2016). Especially when looking at successful leads, derived from fragments, a clear trend toward larger deviation from planarity can be observed (Johnson et al., 2018). Different descriptors exist, that describe the *non-flatness* of a molecule, like fraction of sp^3^ hybridized atoms (Yang et al., 2012), deviation of plane of best fit (Hall et al., 2014) and principle moments of inertia (Sauer and Schwarz, 2003; Aldeghi et al., 2013). Due to the enrichment of aromatic, sp^2^ hybridized, ring systems with halogens, HEFLibs are expected to be relatively flat or rod-like. As expected, the generated library contains a large fraction of molecules that is planar to rod-like with a mean of Fsp^3^ of 0.06 (see Figure 5).

### Similarity of Electrostatic Potential

**Figure 6:**
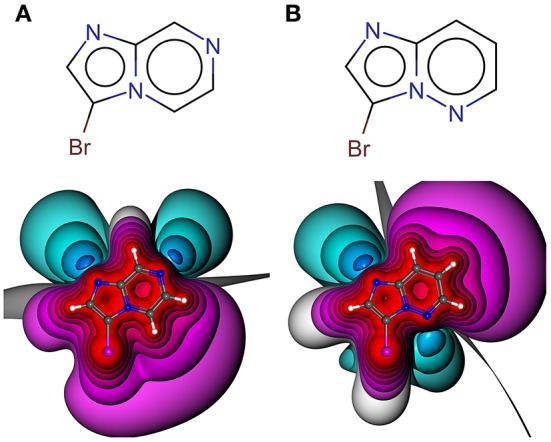
Depicted structures with high structural similarity, but significantly different XB interface: **(A)** 3-bromoimidazo[1,2-a]pyrazine and **(B)** 3-bromoimidazo[1,2-b]pyridazine. Electrostatic potential plot colored from −0.34 au (blue) via zero (white) to +0.34 au (red). Magnitude: V_max, A_ = 0.040 au, V_max, B_ = 0.028 au. Deviation of point of V_max_ from C-X bond vector linearity: Φ(V_max, A_) = 2.0°, Φ(V_max, B_) = 4.6°.

Especially the influence of minor changes in structural topology to the halogen bonding interface, defined as the proximal environment of the σ-hole donor, is commonly underestimated. The explorative character of the library with respect to the binding motif (XB interface) is exemplified in Figure 6, where two constitutional isomers that are part of the novel showcase HEFLib are depicted. They only differ in the position of nitrogen atom 5 or 7. In case of the imidazo[1,2-*b*]pyridazine scaffold (Figure 6B) the lone pair of this nitrogen atom is pointing toward the same direction as the bromine atom and, thus, reduces the extremely tuned magnitude of the σ-hole (V_max_) of the bromine atom in comparison to 3-bromoimidazo[1,2-*a*]pyrazine (Figure 6A).

**Figure 7:**
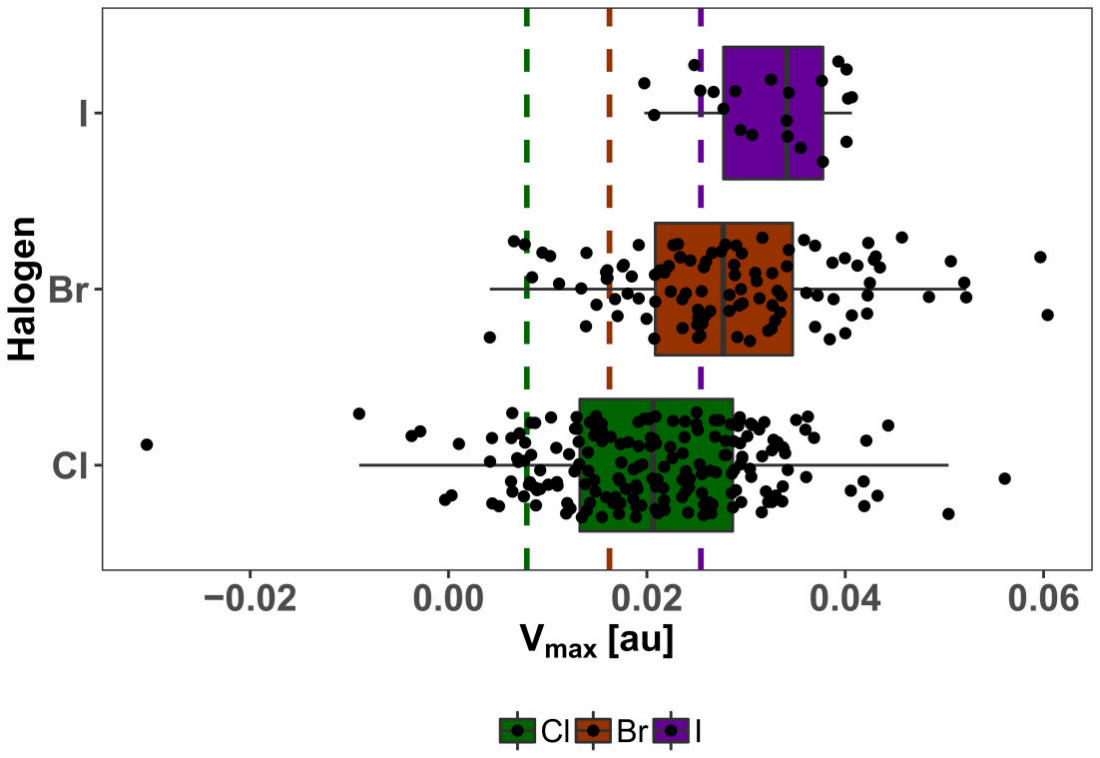
Distribution of V_max_ of neutral isomers in the showcase HEFLib. Color encodes the type of aromatic halogen: Green for chlorine, brown for bromine, and purple for iodine. Same color code is used for reference V_max_ values (dashed lines) of chloro-, bromo-, and iodobenzene. Extreme values are found for zwitterionic fragments.

The strong correlation of V_max_ and the potential adduct formation energy (Politzer et al., 2013; Heidrich et al., 2018; Lange et al., 2019) and its demonstrated usefulness as descriptor for the magnitude of the σ-hole, is the basis for our choice of V_max_ as an important parameter for our diversity assessment, which is centered on the XB interface. As an indicator for the diversity of our showcase HEFLib, we use the distribution of calculated V_max_ values at the classical definition of the electron isodensity level of 0.001 au while including all possible tautomeric states with a net charge of zero. It needs to be mentioned that several of these states, generated by LigPrep/EPIK (Schrödinger Suite), might contribute with a very low probability to the overall distribution of states. In comparison to V_max_ values of chlorobenzene (0.008 au), bromobenzene (0.016 au), and iodobenzene (0.025 au) all median values and also the corresponding first quartile of each halogen type are above the reference (see Figure 7). Remarkably, the range of V_max_ values for the chlorine fragments is larger than that of iodine fragments. Given the higher tunability of iodine in comparison to bromine or chlorine, it should be noted that the spectrum of σ-hole magnitude for iodine compounds in the library was limited by the restrictive cost filter of $5/mg compound.

By evaluating our herein demonstrated showcase library with different target proteins, first hits featuring halogen bonding were successfully identified.

## Conclusion

We aimed for a design strategy with particular focus on the XB interface diversity of halogen-enriched fragments as well as an initial showcase HEFLib with a broad spectrum of targetable features in close proximity to the halogen that can form specific interactions with various elements of the binding site. Parallel to the concept of “privileged structures” (Duarte et al., 2007) that contain binding motifs for targeting specific binding sites, e.g., GPCRs or kinases, our concept brings into focus the molecular interactions of halogen atoms and their proximal environment. By introducing structural diversity into halogenated fragments, exerting significant influences on the predicted V_max_, we obtain a variety of XB interfaces that are able to address a broad spectrum of targets with halogen bonding as key interaction motif.

We have demonstrated that in our generated HEFLib, a diverse halogen bonding interface is also reflected by diverse σ-hole magnitudes of the fragments. We admit that chemical space coverage of our initial showcase HEFLib, especially with regard to three-dimensionality and iodine content, was significantly lowered by limited financial resources in an academic environment. Our library is available for other working groups and can be seen as starting point for further expansion, which is a well-known method of the iterative process of library design and screening (Brewer et al., 2008). Next evolutionary steps of the library are focused on three-dimensionality and even higher tuning values of V_max_. Since approximation of V_max_ values can be efficiently performed using V_max_Pred (Heidrich et al., 2018), we plan to enrich fragments with greater σ-hole magnitudes by custom synthesis using carefully selected building blocks. Based on an *in silico* synthesis approach, vast numbers of potential halogenated fragments can be efficiently proposed and used as a starting point for a selection process as outlined herein. This strategy will significantly increase the probability of establishing stronger halogen bonds as key interactions in early hit finding phases of academic and industrial drug discovery.

The implemented feature tree-like diversity measure can be used for the diversity assessment of halogenated fragments in other projects and is available for other working groups.

## Author Contributions

FB initiated the research. JH implemented similarity measure and performed library filtering. LS and JH performed and evaluated turbidimetric solubility assay. FB and JH prepared the manuscript.

### Conflict of Interest Statement

The authors declare that the research was conducted in the absence of any commercial or financial relationships that could be construed as a potential conflict of interest.
