# Supplementary material for: Embracing the Diversity of Halogen Bonding Motifs in Fragment-Based Drug Discovery—Construction of a Diversity-Optimized Halogen-Enriched Fragment Library
Source: Front Chem. 2019 Feb 18;7:9. doi: 10.3389/fchem.2019.00009 (PMC6387937; doi:10.3389/fchem.2019.00009)
Supplement: Supplementary file 2 [file Data_Sheet_2.pdf]

Supplementary Material

Embracing the Diversity of Halogen Bonding Motifs in Fragment-based Drug Discovery – Construction of a Diversity-optimized Halogen-enriched Fragment Library

Johannes Heidrich[1], Laura E. Sperl[1,3], Frank M. Boeckler[1,2,*]

[1]Lab for Molecular Design & Pharm. Biophysics, Institute of Pharmaceutical Sciences, Department of Pharmacy and Biochemistry, Eberhard Karls Universität Tübingen, Tübingen, Germany

[2] Center for Bioinformatics Tübingen (ZBIT), Eberhard Karls Universität Tübingen, Tübingen, Germany.

*** Correspondence:**
Frank M. Boeckler
frank.boeckler@uni-tuebingen.de

[3] Present Address:
Structural Membrane Biochemistry, Bavarian NMR Center at the Department of Chemistry and Institute for Advanced Study Technical University of Munich, Garching, Germany

1 Supplementary Figures

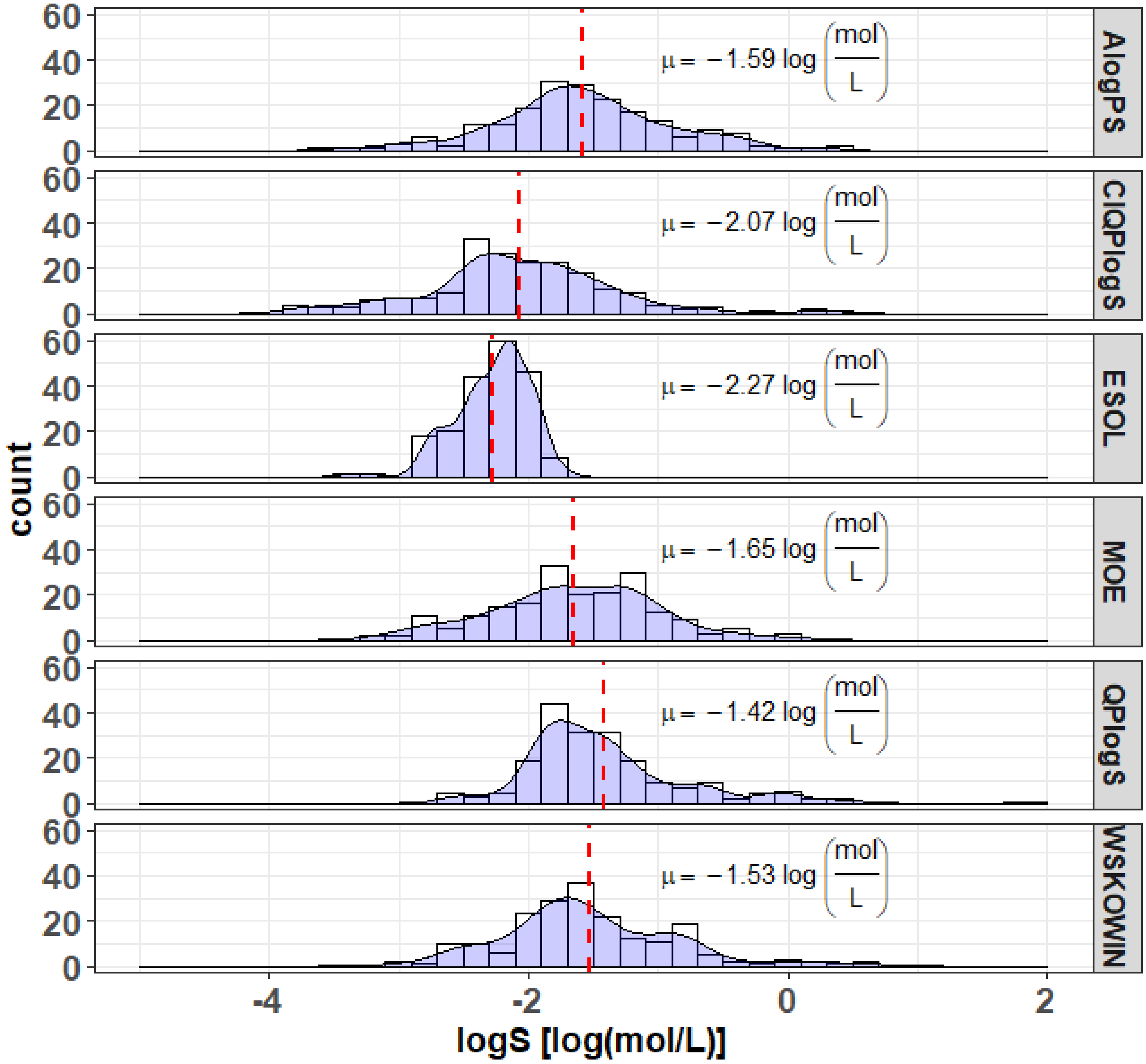

Figure S1. Predicted solubilities and their mean values of showcase HEFLib. Prediction tools and methods: AlogPS, CIQPlogS, ESOL, MOE, QPlogS, WSKOWIN.

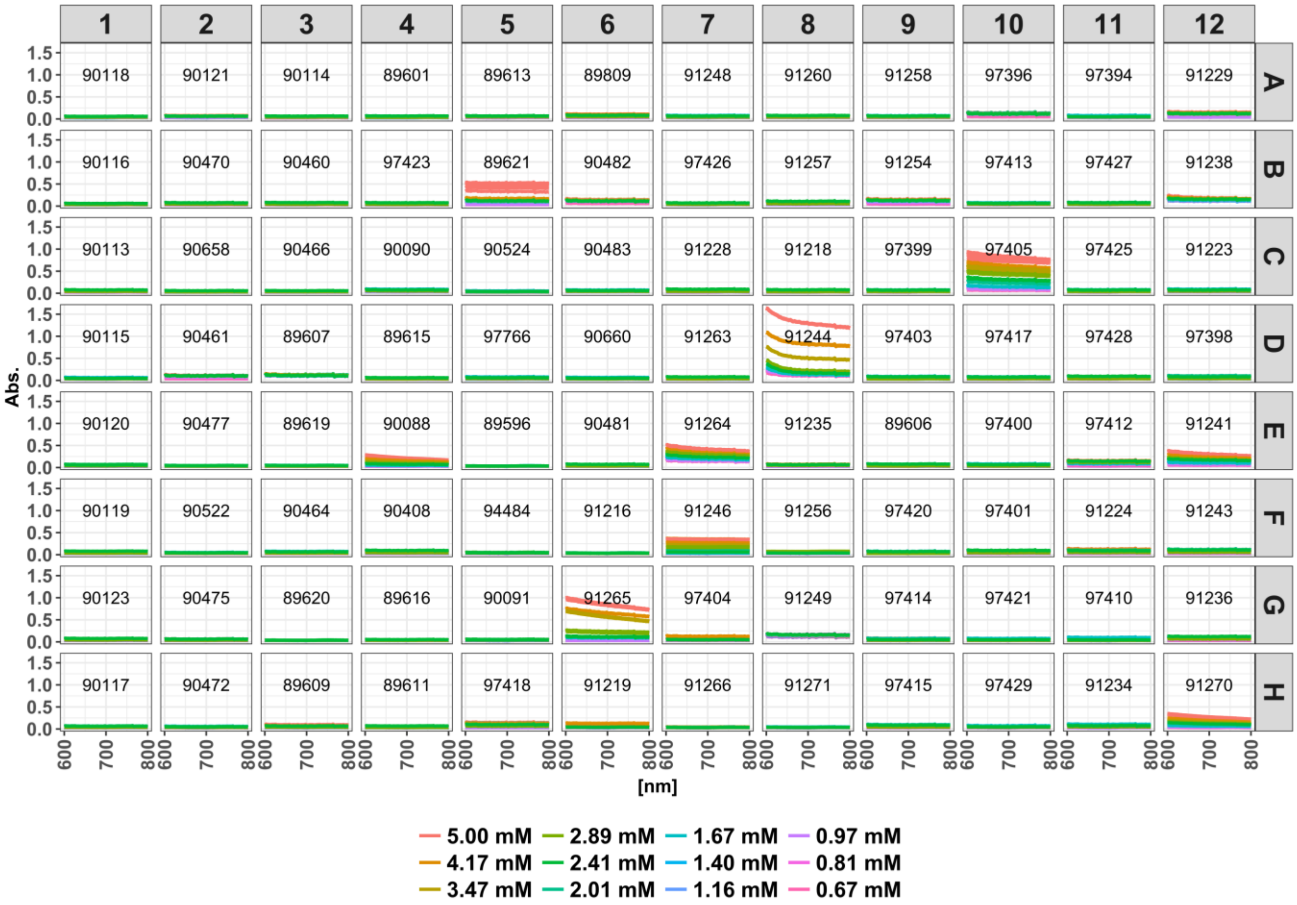

Figure S2. Absorption/extinction spectra for 96 tested fragments from our showcase HEFLib. Scanned wavelength from 600 - 800 nm are shown for concentration levels from 0.67 - 5 mM in 5 % DMSO and buffer (50 mM HEPES pH 7.0, 100 mM NaCl) at room temperature. Readout was done in five kinetic cycles over a time period of 14.6 minutes. Individual fragments are identified by a five-digit number. Fragment 91244 was not completely soluble in 100 % DMSO, thus, the resulting solubility limit remains unclear.

.
